# Claims, liabilities, injures and compensation payments of medical malpractice litigation cases in China from 1998 to 2011

**DOI:** 10.1186/1472-6963-14-390

**Published:** 2014-09-13

**Authors:** Heng Li, Xiangcheng Wu, Tao Sun, Li Li, Xiaowen Zhao, Xinyan Liu, Lei Gao, Quansheng Sun, Zhong Zhang, Lihua Fan

**Affiliations:** Department of Health Management, School of Public Health, Harbin Medical University, Xuefu Road, Harbin, China; School of Applied Foreign Language, Heilongjiang University, Xuefu Road, Harbin, China; Department of Health economic, School of Public Health, Harbin Medical University, Xuefu Road, Harbin, China

**Keywords:** Claims, Compensation, Medical malpractice, Medical litigation

## Abstract

**Background:**

Although China experienced great improvement in their health system, disputes between patients and doctors have increasingly intensified, reaching an unprecedented level. Retrospective analysis of medical malpractice litigation can discover the characteristics and fundamental cause of these disagreements.

**Methods:**

We analyzed medical malpractice litigation data from 1998 to 2011 for characteristics of claims via a litigation database within a nationwide database of cases (1086 cases) in China, including claims, liabilities, injures, and compensation payments.

**Results:**

Among the cases analyzed, 76 percent of claims received compensation in civil judgment (640 out of 841), while 93 percent were fault liability in paid judgment (597 out of 640). The average time span between the occurrence of the injury dispute and closure of claims was 3 years. Twenty-two percent of claims (183 of 841) were caused by injury, poisoning, and other external causes. Seventy-nine percent of claims (472 of 597) were contributed to by errors in medical technology. The median damage compensation payment for death was significantly lower than for serious injuries (P < 0.001; death, $13270 [IQR, $7617–$23181]; serious injury, $23721 [IQR, $10367–$57058]). Finally, there was no statistically significant difference in the median mental compensation between minor injury, serious injury, and death (P = 0.836).

**Conclusion:**

The social reasons for the conflict and high payment were catastrophic out-of-pocket health-care expense in addition to the high expectations for treatment in China. There were no distinguishing features between China and other countries with respect to time of suits, facilities, and specialties in these claims. The compensation for damages in different medical injuries was unfair in China.

## Background

Although China has greatly improved on their health and medicine system, disputes between patients and doctors have increasingly intensified, reaching an unprecedented level. Many factors contribute to the conflicts [1–3] between doctors and patients, including the high health care costs, unreasonable prescription, [1] preventative medicine [4], and unnecessary examinations [3]. Poor investment in the health system and in training and paying doctors in China can lead to medical errors, corruption, and poor communication between doctors and patients [5]. The malpractice disputes occur both in China [6, 7] and other countries [8–11]. Beside eroding the trust [8] between patients and doctors, medical disputes and errors spawned a great deal of economic loss [10]. Several studies have estimated that approximately one RMB in malpractice compensation may be an additional indirect cost leading to hospital losses of 6.7 times more [12]. Though there have been numerous reports or studies on malpractice in China, studies seldom focus on malpractice litigation in China. However, this may be due to the fact that compensation data related to different injuries is not easily available either in China or other countries.

In this study, we investigated the outcomes of malpractice litigation according to structured retrospective reviews of 1086 closed claims from 1998 to 2011 in China. Results from our detailed analyses of patient claims could provide valuable insights into malpractice litigation in China and make significant contributions to both the safety of patients and management of litigation risk.

### The malpractice laws in china

In China, there are three legislative regulations on medical malpractice. The first is the *Rule on the Handling of Medical Accident* from 1987 to 2002. The second is the *Regulations on the Handling of Medical Accident* since 2002. The 2002 regulation replaces the previous 1987 regulation and increases the adequacy and fairness of compensation as well as the procedure for resolving medical disputes [6]. The third regulation is the *Chapter Six Liability for Medical Malpractice of the Tort Law of the People’s Republic of China*, which was adopted by the Standing Committee of the National People’s Congress on December 26, 2009 and became effective on July 1, 2010.

## Methods

### Study sites

We analyzed 1087 claims settled from February 1998 to October 2011 in 29 provinces of China, using a medical malpractice civil litigation database (civil ruling, civil judgment, and civil mediation) from http://www.pkulaw.cn, provided by ChinalawinfoCo. Ltd., Peking University Center for Legal Information. The database is considered of high authority and is distributed and used widely by law professionals throughout China. More importantly, it is admitted by the Supreme People’s Court of China since 2003—Chinese Justice Legal Application Support System (CJLASS) database. All 1086 closed claims in the CJLASS database were allocated to “liability for medical malpractice disputes”. Cases before 1998 were not thoroughly documented with all of the statistical information needed for this study; thus, they were excluded from this study. This study was designed to be a retrospective case study. We intended to find out the characteristics of medical malpractice litigants in China.

We are permitted to access to the database used in our study by ChinalawinfoCo.Ltd. Our study had the approval of the ethics committee of Harbin Medical University of China.

### Statistical analysis

The data sheets, which had been filled out by hand, were electronically entered into a database and verified by a professional data-entry vendor.

We examined characteristics of the court, procedure, appellant, type of medical facility in our samples, percentage of the medical specialties, classification of diseases, injury, degree of liability, and concurrence of causes. We reported the expense in litigation, amount of compensation paid, and days of litigation by using mean value and confidence interval as well as the median and interquartile range. We compared characteristics of errors and injuries, using percentage agreement to determine the probability.

We used the Wilcoxon Rank-Sum test to study bivariate associations of the liability, damage, and mental compensation payment in medical malpractice litigation judgment. Subsequently, analyses were conducted with the use of the PASW Statistics software packages, version 18.0.

For our cross-sectional analysis, we limited our sample to claims paid in 2011, because this was the most recent year with complete data available. Hereafter, the amounts shown are estimated based on $US 1 = ¥RMB 6.34.

### Criteria for coding and definition

Civil ligations have three results in China: “civil ruling”, “civil judgment”, and “civil mediation in court”. A civil ruling is the processing of a result of the procedure problem and does not address the rights and obligations to the defendant-plaintiff. Civil judgment is a verdict by the judge about the malpractice dispute and addresses the rights and obligations to the defendant-plaintiff. Civil mediation is a compromise by the defendant-plaintiff in court.

Hospitals bear the liability in three ways: “liability for breach of contract”, “fault liability”, and “equitable liability”. Liability for breach of contract means that the hospital undertakes the responsibility of a breach of medical service contract with a patient. Fault liability means that the hospital undertakes the liability based on some degree of responsibility. Equitable liability means that although the hospital is not at fault, it provides appropriate compensation for the injured patients as per its current property status.

In our study, “type of medical facility” classified medical facilities into 13 categories according to article three of the Rules for the Implementation of the Regulations on the Administration of Medical Institutions in China.

“Classification of diseases” classified diseases into 23 categories according to the International Statistical Classification of Diseases and Related Health Problems 10th Revision (ICD-10) used by the World Health Organization (WHO).

“Degree of liability” divided liability into five circumstances: “full liability” (hospital accepts 100% liability for patient’s injury), “ultimate liability” (hospital accepts 50%–100% liability), “equal liability” (hospital accepts 50% liability), “secondary liability” (hospital accepts 10%–50% liability), and “minor liability” (hospital accepts <10% liability).

“Concurrence of causes” meant that the hospital’s errors contributed to the patient’s injury. Other causes were “cause of patients and families”, “third person tort and hospital joint infringement”, and “multiple medical institutions joint infringement”.

“Medical error” was classified into “medical technology error” (containing nine common subcategories, e.g., surgery, drugs used, diagnose, treatment related errors, etc.), “medical ethics error” (including infringement of patients’ informed consent or privacy), “medical product error” (means the hospital used defective or unqualified blood and blood products, medical equipment, and drugs, causing the injury), and “medical management error” (including administrative, medical record-related, and risk-related management errors).

We measured “injury” by combining the ten divisions on the basis of disability levels, which were ruled on in the Medical Accident Grading Standard in China (for Trial Implementation), into four categories: “minor injury” (injury below the six disability level; life cannot be managed independently), “serious injury” (one to six disability level; most or part of life cannot be managed independently), “death”, and “emotional injury only”.

## Results

### Relationship between compensation and liability

**Figure 1:**
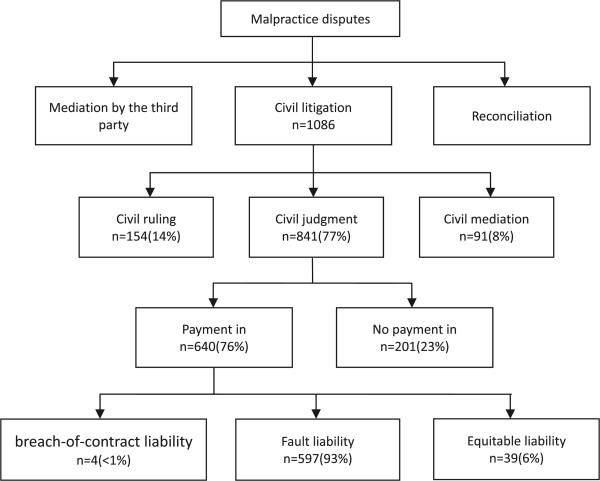
**Overview of the relationship among malpractice disputes, judicial records, outcomes of litigation and form of liability.**

Seventy-seven percent of the litigation cases fell into the judgment category (Figure 1). Most claims received compensation in judgment (640 of 841 [76 percent]) and in mediation (90 of 91 [99 percent]). The outcomes of the payment claims in judgment consisted of three types: breach-of-contract liability (4 of 640 [0.6 percent]); fault liability (597 of 640 [93 percent]), in which the liability was based on some degree of responsibility; and equitable liability (39 of 640 [6 percent]), in which there was a kind of distribution of responsibility and both parties thereto were found to be without fault. Therefore, fault liability is the dominant category in the majority of the claims.

### Characteristics of claims

Seventy-four percent of the claims were solved by an intermediate court (Table 1). Seventy-one percent were closed after the second trial, and 22 percent were closed after the first trial. In 78 percent of cases (n = 844), the appellant—either hospital (31 percent), patient (56 percent), or both (13 percent)—appealed the case.

**Table 1:** **Characteristics of 1086 Claims**

Characteristic		Characteristic	
**Court—no. (%)**	1086	Others	26(3)
Inferior courts	230(21)	**Medical Specialties—no. (%)** ^**¶**^	841
Intermediate court	803(74)	Obstetrics and gynecology	162(19)
Superior court	53(5)	Orthopedic	157(19)
**Procedure—no. (%)**	1086	General surgery	124(15)
The first trial	243(22)	Neurology and neurosurgery	68(8)
Procedure of second Instance	766(71)	Respiratory medicine	52(6)
Retrial process	78(7)	Oncology	37(4)
Appellant—no. (%)*	844	Gastroenterology	36(4)
hospitals	265(31)	Cardiology and cardiac surgery	35(4)
patients	468(56)	Pediatrics	34(4)
both	110(13)	Ophthalmology and otolaryngology	29(3)
**Closure date—no. (%)**	1086	Nephrology	18(2)
1998-2001	74(7)	Urology	13(2)
2002-2005	104(9)	Stomatology	10(1)
2006-2009	485(45)	Others	66(8)
2010-2011	423(39)	**Classification of disease(ICD)—no. (%)** ^**‡**^	841
**Region—no. (%)**	1086		
The west	191(17)	Injury, poisoning and certain other consequences of external causes	183(22)
The midland	572(53)		
The east	323(30)	Pregnancy, childbirth and the puerperium	135(16)
**Expense in litigation—$** ^**†‡**^	**932**		
Expense in litigation per-claim		Diseases of the digestive system	92(11)
Mean	1224	Neoplasms	67(8)
Median	804	Diseases of the respiratory system	58(7)
Plaintiff burden		Diseases of the nervous system	55(6)
Mean	590	Diseases of the circulatory system	53(6)
Median	295	Symptoms, signs and abnormal clinical and laboratory findings, not elsewhere classified	43(5)
Defendants burden			
Mean	639		
Median	263	Diseases of the musculoskeletal system and connective tissue	42(5)
**Time—d.** ^**†**^	**932**		
Time of treatment		Diseases of the genitourinary system	41(5)
Mean	41	Others	72(9)
Median	8	**Degree of liability—no. (%)** ^**‖**^	597
Time from treatment to discover the injury		Full liability	172(29)
		Ultimate liability	185(31)
Mean	311	Equal liability	38(6)
Median	5	Secondary liability	189(32)
Time from the dispute to closure		Minor responsibility	13(2)
Mean	999	**Concurrence of causes—no. (%)** ^**‖**^	597
Median	758	Yes	447(75)
**Type of medical facility—no.(%)**¶	841	No	150(25)
General hospital	558(66)	**Types of concurrent causes— no. (%)**	447
Township hospital	80(10)		
Traditional Chinese Medicine Hospitals	52(6)	Cause of patients and families	344(77)
		The third person tort and hospital joint infringement	76(17)
Specialty hospital	49(6)		
Clinic	48(6)	Multiple medical institutions joint infringement	27(6)
Maternal and Child Health Hospital	28(3)		

*Percentage were calculated with the number of procedure of second instance (n=766) and retrial process(n=78).

^†^the case number of expense in litigation and time included the civil judgment (n=841) and civil mediation (n=91).

^‡^Value are given in 2011 dollars ($U.S.1=¥ 6.34).

^¶^Percentage were calculated with the number of the civil judgment (n=841).

^‖^Degree of liability and concurrence of tort were calculated on the basis of fault liability only (n=597).

Eighty-four percent of the claims were closed between 2006 and 2011, while 39 percent were closed in 2010 or later. Eighty-three percent of the claims took place in the midland and east of China. The average expense of litigation per claim was $1224 (median, $804). The expenses of plaintiff burden, on average, were nearly the same as the defendants’ burden (roughly about $590 vs. $639). The average length of time between the occurrence of the injury dispute and closure of claims was three years (median, two years).

General hospitals were the most frequently sued by the patient in these cases (66 percent), followed by township hospitals (10 percent) and traditional Chinese medicine hospitals (6 percent). Disciplines such as obstetrics and gynecology, orthopedics, and general surgery accounted for the majority of medical specialties involved in the medical malpractice claims (53 percent). Among patients admitted to the hospital, 22 percent of issues were caused by injury, poisoning, and certain other external causes. Sixteen percent were due to pregnancy, childbirth, and the puerperium (16 percent), while 11 percent were caused by diseases of the digestive system.

Among the fault liability with pay claims (n = 597), 66 percent burdened the hospital with full, ultimate, or equal liability. While 75 percent of the claims were concurrence of causes for the injury (n = 447), 77 percent of the concurrent causes were between self-injury by patient or family and hospital infringement for the injury. Secondarily, 17 percent of the concurrent causes were due to either the third person tort or the hospital joint infringements. Finally, six percent of concurrent causes were due to joint infringement by multiple medical institutions.

### Contributing factors

There were four major factors contributing to the injuries: medical technology error (79 percent), medical ethics error (7 percent), medical product error (7 percent), and medical management error (7 percent) (Table 2). Minor injuries (44 percent) and death (34 percent) were the most frequent outcomes in the malpractice claims. Serious injury (20 percent) and emotional injury (2 percent) are the secondary outcomes.

**Table 2:** **Contributing factors associated with medical error by injury**

Type of error		Injury			Total
	Minor injury	Serious injury	Death	Emotional injury only	
**Medical technology error**	**204**	**97**	**165**	**6**	**472(79%)**
Surgery related	93	31	18	0	142(24%)
Drugs used related	25	11	45	1	82(14%)
Diagnose related	23	13	33	1	70(12%)
Treatment related	22	14	29	1	66(11%)
Pregnancy and delivery related	24	15	19	3	61(10%)
Infection related	6	5	6	0	17(3%)
Nursing related	2	3	10	0	15(2%)
Monitor related	5	3	4	0	12(2%)
Anesthesia related	4	2	1	0	7(1%)
**Medical ethics error**	**19**	**8**	**10**	**3**	**40(7%)**
Informant	18	7	10	3	38(6%)
Privacy	1	1	0	0	2(<1%)
**Medical product error**	**28**	**8**	**4**	**3**	**43(7%)**
Blood and blood products	11	4	3	2	20(3%)
Medical equipment	17	4	1	0	22(4%)
Drugs	0	0	0	1	1(<1%)
**Medical management error**	**12**	**7**	**21**	**2**	**42(7%)**
Administrative management	5	6	10	2	23(4%)
Medical record management	3	1	10	0	14(2%)
Risk management	4	0	1	0	5(<1%)
**Total**	**263(44%)**	**120(20%)**	**200(34%)**	**14(2%)**	**597(100%)**

### Compensation payment

There was no statistically significant difference in the median compensation payments between the civil mediation and the civil judgment groups (P = 0.125) (Table 3). Similarly, there was no statistically significant difference in the mental compensation payments between minor injury, serious injury, and death groups (P = 0.836).The median liability compensation payment for fault liability was significantly higher than that of equal liability (P < 0.001; fault liability, $11611 [IQR, $5764–$24894]; equal liability, $2549 [IQR, $1087–$11376]).The median damage compensation payment for death was significantly lower than that for serious injury (P < 0.001; death, $13270 [IQR, $7617–$23181]; serious injury, $23721 [IQR, $10367–$57058]). The maximum payment occurred in minor injury case, fault liability in civil judgment ($628692). In 18 percent of claims (106 of 597), no mental compensation was awarded for injury (minor injury, n = 44; serious injury, n = 25; death, n = 37).

**Table 3:** The compensation payment in medical malpractice litigation, 2011*

Compensation payment	No.	Mean (95% CI)	Median (IQR)	P Value ^†^
**Judgment**				
Civil mediation	90	$16493(11576-21409)	$9108(4466-20482)	0.125
Civil judgment	640	$22508($19005-$26011)	$11113($5315-$24049)	
**Liability**				
Equitable liability	39	$12002($2189-$21815)	$2549($1087-$11376)	<0.001
Fault liability	597	$23318($19621-$27014)	$11611($5764-$24894)	
**Damage compensation**				
Minor injury	263	$15844($10806-$20882)	$7632($3795-$17275)	<0.001^¶^
Serious injury	120	$46662($33456-$59867)	$23721($10367-$57058)	
Death	200	$19565($16686-$22444)	$13270($7617-$23181)	
**Mental compensation**				
Minor injury	263	$3606($2850-$4361)	$1801($535-$4732)	0.836
Serious injury	120	$3159($2240-$4079)	$1812($357-$4541)	
Death	200	$3159($2587-$3730)	$1869($340-$4492)	

*Abbreviation*: *CI* confidence interval, *IQR* interquartile range, *Max* maximum, *Min* minimum.

*Value are given in 2011 dollars ($U.S.1 = ¥ 6.34).

^†^Wilcoxon Rank-Sum test for compensation payment.

^¶^Further Wilcoxon Rank-Sum test for damage compensation differences between minor injury, and serious injury(P < 0.001);As between serious injury and death (P < 0.001),as between minor injury, and death (P < 0.001).

## Discussion

### Conflict between patient and doctor

Research data show that medical disputes increase from year to year. There are a number of reasons for the increase in doctor-patient conflict. With advances in healthcare-related science and technology, patients have unrealistic expectations about treatment, so the physician is more frequently called to answer for any result falling short of patient expectations [10]. This is the common reason both in China and other countries [13], like Italy [10].

In addition, due to poor investment and dramatic marketization of the health system in China, Chinese patients generally spend more than half of their received compensation from medical litigations on their medical costs instead of receiving full coverage from their health insurance companies.

As Liebman and Lancet report, families have had to pay out of pocket up front for healthcare—a phenomenon referred to as “pay or die” [1] or “catastrophic expenses” [5] in China. This special social issue worsens the relationship between doctor and patient.

On the other hand, involved hospitals are also in an unfortunate situation, in which they have to cover expenses for patients’ compensation from medical litigation. This is in contrast with the situation in other countries in which the cost is covered by medical liability insurance companies. In other words, both the patients and the hospitals bear the huge economic burdens of medical malpractice disputes and become direct opposition parties.

### Payment of malpractice claims

After comprehensive analysis of litigation data, we concluded that the majority of claims were generally resolved appropriately through medical malpractice litigation. When combining payment claims for civil judgment and civil mediation together, approximately two thirds of claims (67 percent) resulted in payment. In addition, almost all of the mediated claims in court resulted in compensation payment. However, only a small fraction of claims were solved on monetary compensation without admission of errors by hospitals.

Several previous studies in China and other countries have investigated the outcomes of malpractice claims [14–30]. Their findings vary widely, with 64 to 90 percent of claims judged to result in financial compensation in China [17, 18] and about 25 to 65 percent of claims in the USA [14–16] and other countries resulting in monetary rewards. While informative, each of these Chinese studies contains some flaws—for instance, use of smaller numbers of claims (73 to 356 cases) [19, 20] in the study or narrow focus on a single hospital [19], area [21], or specialty [22, 31], and analysis of a single type of error [23]. A similar situation existed in the USA [24–27, 32], England [28, 29], Japan [30], and France [33]. In addition, most of the Chinese studies did not address the issue of compensation [19, 20, 22, 23]. Our study was designed to avoid these limitations and to conduct a comprehensive analysis. In William B. Weeks’ study, researchers found that financial compensation was made in 65 percent cases [16]. David analyzed 1452 litigation claims and found that, in 55 percent cases, patients received payment [15]. We discovered that a similar proportion of claims (67 percent) received financial compensation in our study. However, a much lower percentage of cases were solved in settlement in court (8 percent) than in William B. Weeks’ study (61 percent). A low percentage of settlements indicates the presence of more intense conflicts between patients and hospitals in China.

### The burden of malpractice litigation

We identified a small difference in the outcomes of litigation for claims, specifically in the duration from the dispute to closure of the litigation. On average, it takes about two years [34] (three years in our study) in China and five years [15] in the USA to complete a litigation, which is longer than the time consumed in the cases of settlement out of court (about 1 year both in China [34] and the USA [16]). These periods are long for plaintiffs to receive final decisions on monetary compensation [15]. However, the prolonged litigation time was a relatively small impacting factor for the defendants in China, where doctors are allowed to work in hospitals normally during the period of litigation. This practice was quite different from that in the USA [15], where doctors involved in lawsuits are not allowed to work during the litigation period. Unfortunately, patients tend to resort to extreme violence in medical disputes in China when they perceive that doctors have poor attitude and lower quality of medical services. Lenient punishments for doctors may partially account for the violence in these medical disputes.

### Reasons for high-risk medical specialties

Our results in type of medical facilities, medical specialties in claims have few distinguishing characteristics compared to other studies. The majority of malpractice disputes in China occur in general hospitals and township hospitals [35]. Obstetrics and gynecology, orthopedics, and general surgery are always the top three medical-risk specialties, both in China [12, 17, 20, 34–37] and in the USA [15, 32]. Similar results were found in Taiwan that malpractice experiences were more frequent in physicians of surgery or obstetrics and gynecology [38]. This phenomenon can be attributed to three factors. First, obstetrics and gynecology deal with newborns or the female reproductive system, and orthopedics handles body movement and work competence. These tend to grab more attention from patients and their families [39]. Second, patients who are admitted to surgery departments often suffer from severe diseases and expect dramatic improvements following a major procedure. Third, surgical procedures are more dramatic and may encounter more risk than other specialties.

### Diseases and liability in litigation cases

We found unexpected outcomes of disease and liability in litigation cases. Injury, poisoning, and certain other external causes were the main issues involved in these claims. The majority of the patients’ injuries had concurrent infringement by other subjects, not only by one hospital. In other words, these medical malpractice claims occurred with greater complexity. In essence, there is no guarantee of completely avoiding or preventing medical risks in China. Furthermore, we found a stark difference in the outcome of degree liability between those settled in court and those settled out of court. The percentage of full liability and ultimate liability was dramatically higher in litigations settled in court (60 percent) than those resolved out of court (8.4 percent) [20]. These results suggest that secondary and minor liability claims are easy to reconcile out of court.

### Errors contribute to injuries

When analyzing the results of errors and injuries, we found that the medical technology errors were certainly the most frequent causes of injuries, rather than errors of staff ethics or management of diseases. Nevertheless, more than half of plaintiffs still questioned the authenticity of medical documents in claims. In fact, some hospitals may forge or change medical documents to cover up their errors. Hospitals hold controlled access to patients’ records and, more often than not, deny patients access to their own medical records. This does underline the challenges in reality that a plaintiff faces when trying to access and acquire evidential information that is crucial for the proof of the claim [40]. In other words, this was the deep-rooted reason for the “malpractice crisis” in China: the lack of a credible system to deal with medical malpractice and to solve related problems in quality of medical care [6].

### Unfair compensation in medical malpractice

We should pay attention to the unjust consequences of various compensation payments for different injuries. The average payment for serious injury ($23721) was more than twice the payment made at death of a patient ($13270). In the Chinese compensation provisions, there is stipulation on the disability compensation but no provision on death compensation. The death compensation stipulation was only introduced in 2010 when the Tort Liability Act, which was responsible for the lower payment in the event of death, was introduced. Additionally, the compensation for moral damages has been legislatively limited or “capped” for plaintiffs in malpractice cases by Chinese legal provisions for compensation [1, 41], which may be the reason for the similarity in compensation among different injuries. These unreasonable and unfair consequences can be mainly attributed to deficiencies or flaws of medical malpractice law in China.

### Limitations

We recognize that our study may contain the following limitations. First, samples were drawn from a legal precedent website involved in medical disputes. These samples may not fully represent malpractice claims nationwide. Other research reported that about 5.4 to 25.3 percent of medical disputes were solved by litigation in all claims [17, 18, 34, 36, 38]. Second, we do not have detailed information on either doctors or patients involved in these litigation cases. Third, the practice of assigning categories in our judgments may not be totally reliable, since arbitrary assignment of categories may not fully represent the complexity of malpractice claims. Fourth, we did not take the liability factor into consideration when comparing compensation amounts in different injuries.

## Conclusion

The social reasons for the conflict and high payment were catastrophic out-of-pocket health-care expense in addition to the high expectations for treatment in China. There were no distinguishing features between China and other countries with respect to time of suits, facilities, and specialties in these claims. The compensation for damages in different medical injuries was unfair in China.
